# Donor template delivery by recombinant adeno-associated virus for the production of knock-in mice

**DOI:** 10.1186/s12915-024-01834-z

**Published:** 2024-02-02

**Authors:** Graham Duddy, Katherine Courtis, Juliette Horwood, Jessica Olsen, Helen Horsler, Tina Hodgson, Sunita Varsani-Brown, Abdullah Abdullah, Laura Denti, Hollie Lane, Fabio Delaqua, Julia Janzen, Molly Strom, Ian Rosewell, Katharine Crawley, Benjamin Davies

**Affiliations:** 1 The Francis Crick Institute, 1 Midland Rd, London, NW1 1AT UK; 2 Transnetyx Inc, 8110 Cordova Rd. Suite 119, Cordova, TN 38016 USA

**Keywords:** CRISPR-Cas9, Knock-in mouse, Gene editing, Adeno-associated virus, Zygote, Electroporation

## Abstract

**Background:**

The ability of recombinant adeno-associated virus to transduce preimplantation mouse embryos has led to the use of this delivery method for the production of genetically altered knock-in mice via CRISPR-Cas9. The potential exists for this method to simplify the production and extend the types of alleles that can be generated directly in the zygote, obviating the need for manipulations of the mouse genome via the embryonic stem cell route.

**Results:**

We present the production data from a total of 13 genetically altered knock-in mouse models generated using CRISPR-Cas9 electroporation of zygotes and delivery of donor repair templates via transduction with recombinant adeno-associated virus. We explore the efficiency of gene targeting at a total of 12 independent genetic loci and explore the effects of allele complexity and introduce strategies for efficient identification of founder animals. In addition, we investigate the reliability of germline transmission of the engineered allele from founder mice generated using this methodology. By comparing our production data against genetically altered knock-in mice generated via gene targeting in embryonic stem cells and their microinjection into blastocysts, we assess the animal cost of the two methods.

**Conclusions:**

Our results confirm that recombinant adeno-associated virus transduction of zygotes provides a robust and effective delivery route for donor templates for the production of knock-in mice, across a range of insertion sizes (0.9–4.7 kb). We find that the animal cost of this method is considerably less than generating knock-in models via embryonic stem cells and thus constitutes a considerable 3Rs reduction.

**Supplementary Information:**

The online version contains supplementary material available at 10.1186/s12915-024-01834-z.

## Background

The discovery of the type II CRISPR-Cas system [1], a bacterial adaptive immune system capable of introducing double-strand breaks (DSBs) into DNA in a sequence-specific manner [2, 3], has initiated a revolution in the field of gene editing and gene targeting [4–7]. Earlier programmable nucleases such as zinc finger nucleases [8] and TALENS [9] were capable of site-specific DNA cleavage, but their design and molecular construction were challenging. In contrast, CRISPR-Cas9 can be harnessed with relative speed and ease of use, using simple design rules. In addition, numerous web-based design tools are available [10, 11] and CRISPR-Cas9 reagents are commercially available from several companies. Consequently, simple manipulations of the genome can be achieved with relatively high efficiency by delivery of CRISPR-Cas9 reagents via either plasmid DNA [12], RNA [13] or ribonucleoprotein [14], and these techniques have rapidly become commonplace in laboratories throughout the world.

The introduction of CRISPR-Cas9 reagents alone into a target cell or embryo can be used to disrupt gene function via the introduction of disruptive indels or in cis deletions between two adjacent cut sites, as a result of the non-homologous end-joining repair pathway. If an appropriately designed homologous donor template molecule is co-introduced with the CRISPR-Cas9 reagents, repair of the DSB can occur via homology-directed repair and specific mutations can be introduced or repaired. With respect to the production of genetically modified animal models, CRISPR-Cas9 reagents and donor templates can be introduced directly into the fertilised zygote by either microinjection [15] or electroporation [16, 17]. Both methods, however, have associated limitations and challenges.

Microinjection of CRISPR-Cas9 reagents into the cytoplasm or pronucleus has proven successful for the generation of knock-out mutations [13] or in combination with small single-stranded oligodeoxynucleotides (ssODN) [15] or larger double-stranded DNA (dsDNA) templates [18] for the production of knock-in mutations. Microinjection techniques are technically challenging and require highly trained staff and dedicated microinjection and microscopy equipment. This is not always available to laboratories intending to access the potential of CRISPR-Cas9 gene editing. To overcome this hurdle, electroporation of zygotes has proven successful as an alternative delivery route for CRISPR-Cas9 reagents [16, 17, 19]. Electroporation is an attractive option owing to its relative ease, high throughput and the non-specialised laboratory equipment required. However, the electroporation delivery route is not ideal for the introduction of more complex large donor molecules and, despite a few isolated reports [20], the technique has been used mainly for the delivery of small ssODN templates.

For larger insertions, where delivery by microinjection is required, numerous formats of donor template have been employed. Examples include large dsDNA-based donor molecules including circular plasmids [21], CRISPR-Cas9 cleavage-released constructs [22] and linear or PCR-generated dsDNA products [23]. Arguably the most promising breakthrough has been generating donor templates as long single-stranded DNA (lssDNA) rather than dsDNA [24], which led to increased targeting efficiencies in many facilities [25]. This technique is still limited by size, not only by the inverse relationship between HDR efficiencies and increasing donor insert size, but also by the challenges of production, stability and quality control of the required lssDNA molecules.

An alternative approach to template delivery is the use of recombinant adeno-associated virus (rAAV). Naturally occurring serotypes of AAV are able to transduce preimplantation mouse embryos and deliver the single-stranded DNA (ssDNA) genome to the nucleus at high efficiency [26]. Successful gene editing of the mouse zygotes was first achieved by delivery of CRISPR-Cas9 machinery and donor templates on two separate rAAVs [26]. This strategy was further refined through electroporation of CRISPR-Cas9 reagents as ribonucleoprotein and transduction with an rAAV encoding a donor template molecule which achieved high targeted efficiencies in both mouse [27, 28] and rat embryos [27].

Prior to these technical improvements in template delivery, a robust pipeline for large knock-ins necessitated the intermediate step of gene targeting in embryonic stem (ES) cells, with the actual model then being produced by generating chimeras of correctly targeted ES cells with wild-type host embryos. With a cargo capacity of approximately 4.7 kb, rAAV template delivery extends the types of alleles that can be directly generated in the zygote without requiring ES cells.

Given the advantages of rAAV template delivery with respect to size and the avoidance of the technically challenging and labour-intensive microinjection and ES cell targeting, we sought to introduce an efficient and robust CRISPR-Cas9 rAAV workflow into our own facility. Here, we present that work, including complete production data from 13 gene editing projects across 12 distinct genetic loci. We examine the efficiency of this approach by assessing embryo survival, birth rate, targeting efficiency and germline transmission. We also compare this method with the traditional ES cell-based approach with respect to the animal cost of genetically modified mouse production.

## Results

**Fig. 1:**
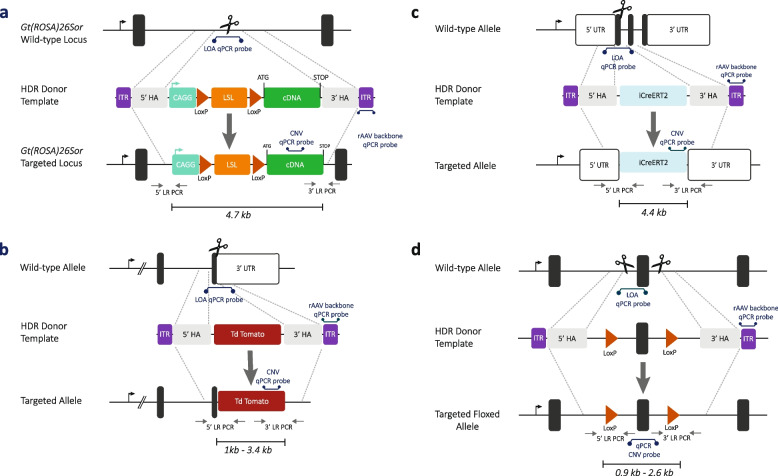
Examples of knock-in alleles generated and their genotyping. **a** Simple transgene insertions (CAGG-LoxP-STOP-LoxP-cDNA knock-in), **b** simple 3′ UTR knock-in of a fluorescent reporter, **c** simple functional cassette knock-in and **d** LoxP flanked conditional allele. 5′ and 3′ long range (5′ LR and 3′ LR) are designed such that one primer is specific to regions external to the homology, while the second primer is specific to the intended inserted cassette. qPCR genotyping assays included loss of allele (LOA) designed against the wild-type allele; copy number variation (CNV) qPCR is designed against the insert and to the rAAV inverted terminal repeats

The rAAV donors described in this study encompass a variety of alleles: integration of functional cassettes such as Cre recombinase, rtTA or fluorescent reporters, conditional overexpression cassettes, conditional Knock-in and floxed alleles (Fig. 1). The full production data for 13 individual mutant mouse projects encompassing 12 distinct genetic loci generated using rAAV donor template delivery and CRISPR-Cas9 ribonuclear protein (RNP) electroporation are shown in Additional file 1: Tables S1 and S2, with a summary table for each generated allele shown in Table 1.

**Table 1:** Summary production statistics for the 13 projects up to the generation of validated F0 founder knock-in mice

Project	Allele type	Insert size (kb)	Homology arm length (kb)	Embryos transferred	Pups born	Birth rate	No. of F0 mice genotyped	No. of F0 mice positive by qPCR	No. of validated F0 knock-in mice (% targeting)	% knock-in of total F0 mice genotyped	% knock-in of qPCR-positive F0 mice
GMAF	Complex	2.3	0.4	171	34	19.9%	34	14	5	14.7%	35.7%
GMAG	Simple	2	1	110	9	8.2%	9	2	2	22.2%	100.0%
GMAJ	Simple	2.6	0.4	335	51	15.2%	51	21	19	37.3%	90.5%
GMAL	Simple	4.7	0.4	107	18	16.8%	9	9	4	44.4%	44.4%
GMAM	Simple	4.7	0.4	205	20	9.8%	11	6	4	36.4%	66.7%
GMAN	Simple	3.4	1	278	26	9.4%	26	6	4	15.4%	66.7%
GMAR	Complex	0.9	0.4	398	38	9.5%	38	1	1	2.6%	100.0%
GMAW	Simple	1	0.4	150	34	22.7%	34	9	4	11.8%	44.4%
GMAZ	Complex	2.6	0.6	138	31	22.5%	31	3	2	6.5%	66.7%
IRCK	Complex	1.8	0.6	185	31	16.8%	31	13	8	25.8%	61.5%
IRCU	Simple	3.9	0.9	87	7	8.0%	7	3	3	42.9%	100.0%
IRDB	Simple	4.4	1	278	26	9.4%	25	9	6	24.0%	66.7%
RLEB	Simple	1.7	0.5	167	21	12.6%	21	9	7	33.3%	77.8%

**Fig. 2:**
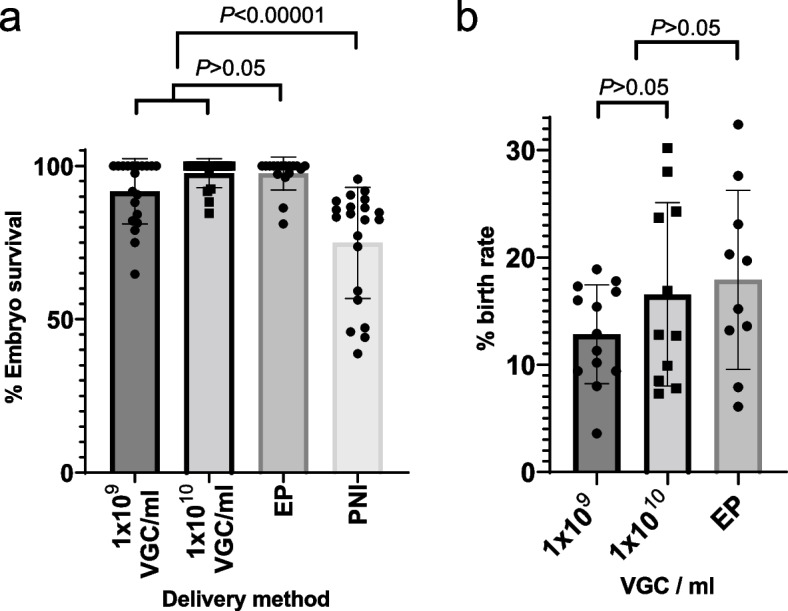
Embryo survival and birth rate. **a** Embryo survival rates are shown for the two titres of rAAV inoculation used (1 × 10^9^ VGC/ml: *n* = 20; 1 × 10^10^ VGC/ml: *n* = 19) and for simple electroporation (EP; *n* = 17)) and pronuclear microinjection (PNI; *n* = 21) of CRISPR-Cas9 reagents as a comparison. **b** Birth rate following embryo transfer of manipulated embryos at the two titres (1 × 10^9^ VGC/ml: *n* = 13; 1 × 10^10^ VGC/ml: *n* = 11) of rAAV used, alongside simple electroporation (EP; *n* = 10) experiments without rAAV template delivery. Statistical analyses of pairwise comparisons between two groups are shown

After the initial ex vivo work to determine suitable titres, all rAAV transductions were performed side by side at both 1 × 10^10^ viral genome copies (VGC)/ml and 1 × 10^9^ VGC/ml titres. A high rate of embryo survival was observed for both higher (97.95%) and lower (91.59%) viral titres (Fig. 2a). We found no difference when comparing the rates of embryo survival following delivery of repair template via rAAV infection and CRISPR-Cas9 RNP electroporation with CRISPR/Cas9 RNP electroporation alone, suggesting no apparent toxicity results from rAAV inoculation (*z* =  − 1.16; *P* = 0.246; Fig. 2a; Additional file 1: Table S3). In addition, the high rates of embryo survival seen with rAAV transduction/CRISPR-Cas9 electroporation contrast with the more modest survival rates observed following contemporaneous pronuclear microinjection of gene editing reagents from unrelated experiments (*z* =  − 18.26; *P* < 0.0001; Fig. 2a; Additional file 1: Table S3). The delivery regime via electroporation and rAAV transduction, even at high titre, is thus well tolerated and avoids the embryo loss that typically occurs following the damage that can be caused by the mechanical act of microinjection. In agreement, the birth rate when averaged across all experiments was 13.4% for the lower titre and 14.1% for the higher titre with no significant difference between titres (*β* = 0.11; *P* = 0.414; Fig. 2b), when assessed on a per allele basis. The birth rates observed were also comparable with those reported following electroporation of CRISPR/Cas9 reagents without rAAV [29] (*z* =  − 1.08; *P* = 0.28; Fig. 2b; Additional file 1: Table S3), again supporting the conclusion that the inoculation with rAAV did not have any adverse effects on embryo survival or subsequent development.

With respect to the targeting efficiency, an initial screen based on genomic qPCR using a primer and probe set specific for the targeted allele to assess the number of integrating copies (copy number variation (CNV) qPCR) and correlating this result with a loss-of-allele (LOA) qPCR designed to detect the unmodified allele was used [18] (Fig. 1). In addition, a qPCR designed against the inverted terminal repeat sequence of the rAAV backbone was used to detect random, on-site or off-site integration of the viral vector genome, but no putative founder mice were generated which scored positive for this assay, suggesting integration events of this kind were rare.

This approach allowed the swift elimination of F0 progeny displaying minimal or no putative targeting as well as mice in which multiple copies of the targeting vector had integrated into the genome, allowing further genotyping effects to be focused. Putative positive F0 mice were then analysed for correct integration of the targeting vector using PCR amplification across the 5′ and 3′ homology arms. Founders scoring positive for both assays were then validated by Sanger sequencing of the 5′ and 3′ homology arm PCRs.

**Fig. 3:**
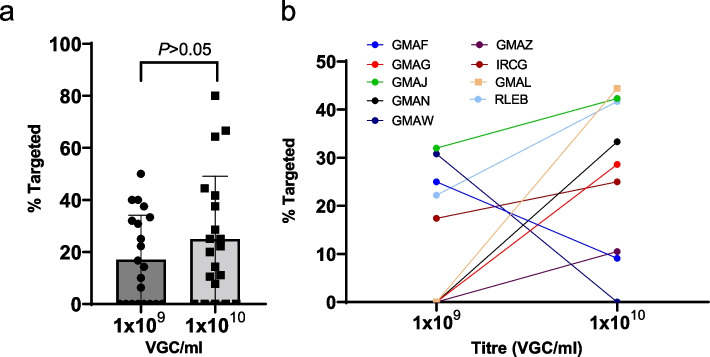
Targeted knock-in efficiency. **a** Targeted knock-in efficiency at the F0 generation is shown for the two titres of rAAV inoculation used with each datapoint representing a specific embryo electroporation/transduction session (1 × 10^9^ VGC/ml: *n* = 21; 1 × 10^10^ VGC/ml: *n* = 20). **b** Targeted knock-in efficiency on a per allele basis (each allele has a unique four-letter code) showing the efficiencies obtained at the two different rAAV titres used

Treating each delivery session independently, targeting efficiencies varying from 0 to 80%, with a mean of 25.0% when a viral titre of 1 × 10^10^ VGC/ml was used and 17.1% when a viral titre of 1 × 10^9^ VGC/ml was used. When considering the effect of rAAV titre for specific alleles, although there was no significant difference in the mean targeting rate between the two viral titres (*β* = 0.55; *P* = 0.12; Fig. 3a), it was apparent that the majority of the targeting experiments (7/9) performed at both titres led to a higher rate of targeting when the higher titre of 1 × 10^10^ VGC/ml was used (Fig. 3b). Interestingly, one experiment (GMAR) failed to yield targeted F0 founders at both 1 × 10^9^ and 1 × 10^10^ VGC/ml but yielded a single founder when tested at 1 × 10^11^ VGC/ml. For the generation of each targeted allele, several delivery sessions were performed. Examining the targeting efficiency from the perspective of the targeted allele and thus ignoring differences in the viral titre used or other experimental details, targeting efficiency varied from 2.6 to 44.4%, with a mean rate of 24.4% (Table 1).

**Fig. 4:**
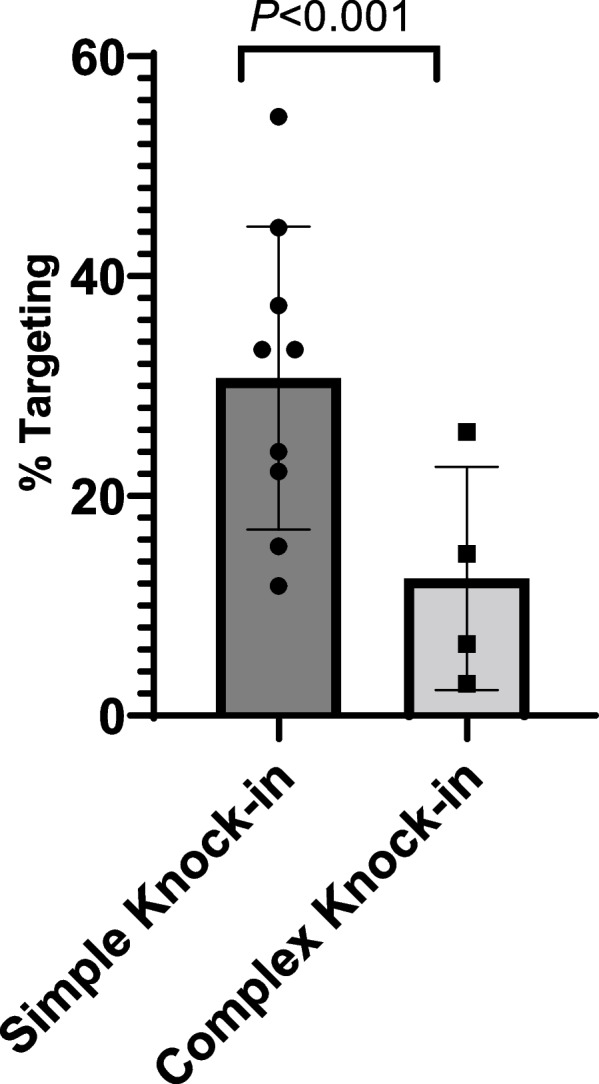
Targeting efficiencies per allele. The effects of the type of allele on targeted knock-in efficiency. Simple knock-in (*n* = 9) are insertions of exogenous sequence whereas complex knock-ins (*n* = 4) involve both exogenous and endogenous sequences, for example, floxed alleles. The individual data values are shown in Table 1

When considering the type of engineered allele, these were assigned into two categories based on the complexity of the insertion. Where the insertion was entirely exogenous sequence, for example, in the case of a fluorescent reporter gene, a Cre recombinase, a reverse tetracycline transactivator, a simple cDNA or a large promoter-cDNA, these alleles were assigned as being simple insertions (insertion sizes 1.0–4.6 kb; Fig. 1a–c). Where the insertion was a mix of exogenous sequence but also contained significant endogenous sequence, for example, in the case of a floxed or a conditional knock-in allele, these alleles were assigned as being complex insertions (insertion sizes 0.9–2.6 kb; Fig. 1d). There was a significant difference (*z* =  − 3.38; *P* = 0.0007) between these groups, with complex insertions revealing a lower average rate of targeting than more simple insertions (Fig. 4).

**Fig. 5:**
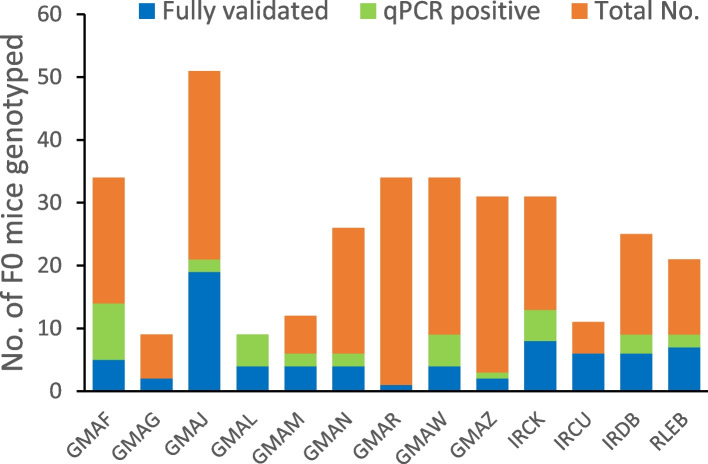
Genotyping effects. Overlayed histograms for each engineered alleles showing the number of pups born (orange), the number validated by Q-PCR to suggest successful targeting (light green) and the number of fully validated founders by PCR and full sequence confirmation (blue)

The approach to genotyping taken allowed us to conclude whether relying simply on LOA/CNV qPCR assays is a reliable measure of determining correct targeting. On a per-targeted allele perspective, between 35 and 100% of these initially identified pups were subsequently found to be correctly targeted, when further validated by PCR and sequencing for targeted integration (Table 1). The data suggest that the use of qPCR is an informative indicator of correct gene targeting, but cannot be relied upon alone (Fig. 5).

Up to 4 independent F0 founders were bred with wild-type C57BL/6 J mice to obtain F1 heterozygous for the engineered allele. Of the 13 projects reported, 12 were taken forward for breeding (1 project was not bred to F1 as the allele was secured by parallel gene targeting activities in embryonic stem cells). All 12 projects led to the generation of F1 mice which were validated by the LOA/CNV assays, 5′ and 3′ PCR over the homology arms and sequence validation by Sanger or long-read sequencing of the screening amplicons (Table 2).

**Table 2:** Germline transmission data for the 12 projects bred to the F1 generation

Project	No. of founders bred	No. of founders producing litters	No. of founders which transmitted a validated F1	No. of founders which transmitted a validated F1 in 1st litter	No. of F1s from transmitting founder tested	No. of heterozygous F1
GMAJ	4	4	3	3	24	9
GMAF	3	3	3	3	18	8
GMAL	4	3	2	2	27	6
GMAM	2	2	1	1	18	4
IRDB	1	1	1	1	10	5
RLEB	3	3	3	2	27	15
IRCU	3	3	3	3	21	5
GMAG	2	2	2	2	17	11
GMAN	4	4	1	1	11	11
GMAZ	2	2	2	1	18	9
GMAW	3	3	2	2	15	5
GMAR	1	1	1	1	3	2
Totals	31	30	23 (76.7%)	21 (91.3%)	207	90 (43.5%)

Across all of the projects, a total of 31 independent founders were bred, 30 of which yielded offspring, and 23 of these generated F1 mice a subset of which harboured the expected engineered alleles (76.7%). Twenty-one of these 23 transmitting F0 founders yielded validated F1 mice harbouring the expected engineered allele in the 1st generation (91.3%). In total, 207 F1 mice were born from the transmitting F0 founders, and 90 of these F1 mice harboured the expected engineered allele (43.5% germline transmission), which is close to the expected Mendelian frequency of 50%, assuming a non-mosaic heterozygous knock-in founder. Despite the high rate of germline transmission, there was a small deviation from this Mendelian frequency (*χ*^2^ = 3.52; *P* = 0.061) suggesting a degree of mosaicism in the founder mice generated using the rAAV and CRISPR-Cas9 electroporation approach.

**Fig. 6:**
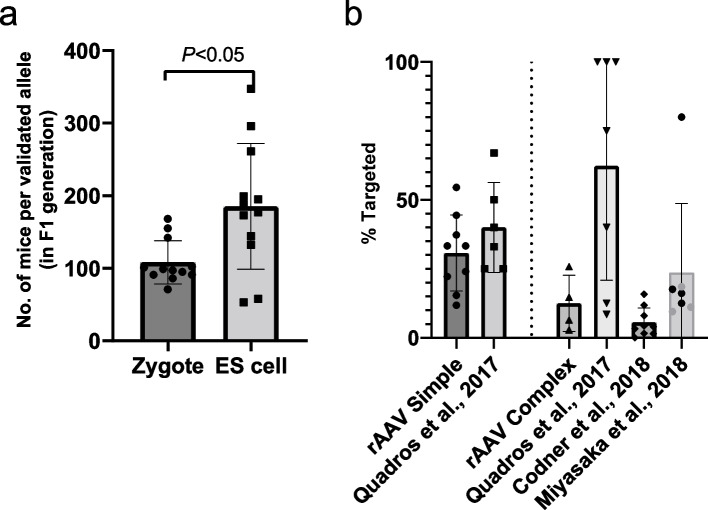
Technology comparisons. **a** The average animal usage is shown for genetic manipulation of the zygote using rAAV template delivery/CRISPR-Cas9 electroporation (Zygote; *n* = 12) compared with contemporaneous projects performed using ES cell targeting and blastocyst injection (ES cell; *n* = 12). The numbers include the F1 generation. **b** Comparison of the per allele targeting efficiencies for simple and complex projects (rAAV Simple: *n* = 9; rAAV Complex: *n* = 4; this study) versus previously published data using lssDNA microinjection (Quadros et al., 2017; simple: *n* = 6; complex: *n* = 4 [25]; Codner et al., 2018: *n* = 9 [30], Miyasaka et al., 2018: *n* = 7 [20] In this latter study, data points from electroporation of lssDNA are shown in light grey)

We next compared the animal cost of direct modification of zygotes using rAAV and CRISPR-Cas9 electroporation across the 12 projects that yielded validated F1 mice, taking into consideration the number of embryo donors, the embryo transfer recipients, the F0 founders generated and their F1 progeny. These numbers were compared to 12 contemporaneous projects which were performed via gene targeting in ES cells, blastocyst injection of correctly targeted ES cells, chimera production, breeding and confirmation of germline transmission in the F1 generation. Alleles of similar size and complexity were chosen for this comparison (Additional File 1: Table S4); however, the comparison that is made here concerns only the animal cost of the model generation with the targeting efficiency in ES cells not considered a factor. Once targeting ES cells are generated, the total animal cost in converting these ES cell clones into transmitted F1 heterozygous mice was calculated. In total, the 12 projects completed by direct modification of the zygote resulted in the use or generation of 1298 mice, with an average of 108 mice per project/engineered allele. This compares favourably (*z* =  − 2.45; *P* = 0.014) with the traditional ES cell route: from the twelve randomly selected successfully ES cell targeting experiments, the total number of mice used or generated was 2223, with an average of 185 mice used per project (Fig. 6a).

For the ES cell route, a large excess of mice (relative to the production data for the rAAV/CRISPR-Cas9 electroporation zygote manipulations) were observed in two areas of production. Firstly, the number of donor females required for a successful ES cell microinjection experiment was found to be considerably higher than for the zygote route (73.8 vs 33.3; *z* =  − 2.92; *P* = 0.035) (Additional file 1: Table S4), presumably due to the relatively low yield of high-quality blastocysts suitable for ES cell microinjection when compared with the typical yield of fertilised zygotes. The second area relates to the founder breeding, with a larger number of chimera breeding pairs needed with the ES cell route when compared to founder breeding pairs from the zygote route (8.6 vs 3.2; *z* =  − 3.09; *P* = 0.002) (Additional file 1: Table S4).

## Discussion

We have successfully established a robust pipeline for the generation of genetically modified mouse lines by using CRISPR-Cas9 targeting, delivering the repair template as an rAAV. Our production data suggest that this technology can provide an efficient route for the production of Knock-in alleles harbouring up to 4 kb of exogenous sequence. Furthermore, the methodology requires no microinjection procedures and is thus less reliant on highly trained staff and is potentially more amenable to a high-throughput production pipeline. Our data further suggest that a single electroporation session using these parameters and concentration would be successful in approximately 90% of cases, potentially allowing the production to be scaled appropriately to minimise animal usage further.

Avoiding microinjection by using viral delivery of repair templates is beneficial from an animal usage perspective as, in our hands, less physical damage occurs to the embryo and a higher number survive. Indeed, we found that a viral titre of 1 × 10^10^ VGC/ml was the most effective at achieving targeted mutagenesis, and the use of this concentration was without detriment to embryo survival. A previous study also concluded that higher titres correlate with higher knock-in efficiencies but found that high titres might affect embryo viability [28]. Potentially, the purity of the viral preparation may influence this latter effect, but we found no evidence for any negative impact on embryo survival when using rAAV at a high titre.

In addition, we found that the rAAV titre had no influence on the birth rate following the embryo transfer of the manipulated embryos, and the birth rates obtained were entirely comparable with those reported from electroporation of CRISPR-Cas9 reagents alone [29], confirming that the rAAV treatment of the embryos is without consequence with respect to embryo development.

Despite the high efficiency of targeting observed in our study, rAAV does have a packaging limit of around 4.7 kb, which, with standard homology arms lengths of around 400 bp, restricts the size of knock-in alleles that can be generated to approximately 4 kb using this technology. In contrast, the classical delivery route of pronuclear microinjection can be used to achieve considerably larger knock-in alleles, although delivering large multi-kilobase targeting vectors remains technically challenging, both with respect to preparing a high-quality template and the physical microinjection procedure. It is worth noting, however, that embryo survival following microinjection can also be improved by microinjection at negative capacitance [31].

With respect to the genotyping methodologies, our reliance on LOA/CNV qPCR analysis as a first-pass genotyping approach has allowed for rapid elimination of non-targeted pups, providing a more efficient use of subsequent more elaborate PCR- and sequencing-based genotyping as well as reducing the number of putative founder mice needed to be kept. The data confirms that one cannot rely solely on qPCR-based approaches to confirm gene targeting.

Contrary to what one might expect, we observed a good rate of knock-in efficiency over a wide range of insertion sizes, implying that the methodology can be applied efficiently across all sizes that can be accommodated within the rAAV cargo capacity. There was, however, a large variation in targeting efficiency observed between projects, which presumably relates to the chromosome location, the specificity of the regions of homology used for gene targeting and the efficiency of the CRISPR-Cas9 nucleases. Developmental consequences of a gene’s loss-of-function can contribute to low birth rate and thus targeting efficiency, but in the case of the 12 loci being addressed in this study, none of the regions was associated with any embryonic lethality. The only factor that clearly impacted the targeting efficiency was the inclusion of regions of homology within the inserted gene sequence—the simplest example of this being floxed alleles, where the inserted sequence is defined by the two loxP sites flanking the critical exon region to be conditionally deleted. The homology that exists between these two loxP could potentially reduce the number of correctly targeted founders, i.e. founders that have the two integrated loxP sites. In addition, these projects require two sites of CRISPR-Cas9 activity in cis (Fig. 1d) which can result in a high level of deletion between the cut sites, potentially reducing the efficiency of targeted integration of the template.

Comparing our production data with the results from previously published lssDNA studies [20, 25, 30] suggests that rAAV delivery of repair templates in combination with CRISPR-Cas9 electroporation produces very comparable results for simple insertion knock-ins (Fig. 6b). With respect to complex alleles, e.g. floxed alleles, the comparison is somewhat complicated by a large discrepancy in reported efficiencies in published datasets [20, 25, 30] (Fig. 6b). Potentially, this reflects the technically challenging aspects of lssDNA synthesis, quality control and microinjection which the rAAV delivery route would potentially alleviate. The discrepancy in efficiencies may also reflect differences in the rigour of genotyping as two of the studies used in this comparison did not perform LOA/CNV qPCR analysis [20, 25].

With respect to lssDNA synthesis, it is known that mutations can occur during the synthesis, depending upon the method used, which can lead to the incorporation of mutations within correctly targeted alleles [30]. It remains undetermined how accurate the rAAV virus production is in this regard, but in our study, we did not find any examples of point mutations within the designed alleles that could be attributed to template synthesis errors.

Ectopic integration of rAAV constructs either randomly in the genome or at off-target CRISPR sites remains a concern as well as concatemer recombination at the on-target CRISPR site [32, 33], but such events would be excluded by the qPCR assay designed to assess the copy number of targeted events. The simple insertion of the rAAV construct into the on-target site (rather than by homologous recombination) has also been reported [32], but founders with such a configuration would be excluded by the targeted PCR screen over the homology regions. In this study, we did not characterise any incorrectly targeted founder mice in detail, so we are unable to conclude on the absolute occurrence of these various phenomena but are confident that the combination of screening approaches used in our study is able to detect aberrantly targeted founder mice.

Genetically modified mouse models generated with nuclease reagents delivered to the zygote are known to be mosaic [34], due to the persistence of the nuclease after the first cleavage events in the developing embryo. The mosaicism of a genotyped founder mice can thus explain why, on occasion, the targeted allele is not found in Mendelian proportions in the F1 generation or can even lead to a situation where a correctly genotyped founder mouse does not transmit the targeted allele. Our data across the 12 projects bred to the F1 generation suggests mosaicism is present but is potentially a minor concern, with over three quarters of the founders transmitting the expected engineered allele.

Our data further suggest that the total number of mice required in the production of the average validated engineered allele at the F1 generation is significantly lower via direct manipulation in zygotes as opposed to the ES cell-based classical gene targeting and blastocyst injection route. Clearly, there is a wide discrepancy in mouse usage between different projects based on the need for multiple repeats due to, for example, a low efficiency of targeting. However, the data does suggest that, from a 3Rs perspective, for those alleles which can be generated within the limitations of rAAV packaging size, replacing the ES cell targeting route with in-zygote targeting via electroporation/rAAV transduction would greatly reduce the number of mice required for the production of genetically modified mouse models.

## Conclusions

Delivery of knock-in templates via rAAV, in combination with CRISPR-Cas9 delivery via electroporation, is a robust methodology for the generation of simple and complex knock-in mouse models. The knock-in efficiencies obtained using this technology are comparable to lssDNA microinjection but, importantly, rAAV is able to accommodate larger insertion sizes that can typically be synthesised as lssDNA. Critically, the method avoids the need for technically demanding microinjection and provides a route for Knock-in model generation without this skill set or equipment. The generation of knock-in alleles within the zygote avoids the need for ES cell targeting and microinjection and comes at a reduced animal cost.

## Methods

### Generation of gene editing reagents

Donor template plasmids were constructed using a combination of commercial DNA synthesis (GeneWiz Germany GmbH and VectorBuilder GmbH), conventional cloning and Gibson assembly [35]. The plasmid constructs were then packaged into AAV serotype 1 either by VectorBuilder GmbH or in-house using the packaging plasmids, pAAV2/1 (Addgene #112,862) and pHelper (Agilent Technologies), as previously described [36], but with the production scaled down to 3 × 10 cm^2^ dishes. Post-packaging, rAAV titres were determined as previously described [37], using a QuantStudio3 (Thermo Fisher) using PowerUp SYBRgreen master mix (Thermo Fisher).

CRISPR-Cas9 target sites were designed (Additional File 1: Table S5) and assessed for predicted on-target activity and specificity using the WGE [10] and the CRISPOR [11] algorithms. Synthetic RNA reagents were purchased from Integrated DNA Technologies and Merck. When required, 200 μM of crRNA was annealed with 200 μM of tracrRNA with 4 μl of duplex buffer (Integrated DNA Technologies) in a total volume of 10 μl for 5 min at 95 °C and allowed to cool to room temp. RNP complexes were generated using 100 μM of pre-annealed cr/tracrRNA or 100 μM of sgRNA, complexed with 7.7 μM Cas9 protein (Cas9 Alt-R™ Hifi V3, Integrated DNA Technologies or PURedit™, Merck) and stored on ice prior to electroporation.

### Delivery of gene editing reagents

Immature C57BL/6 J females (Jackson Laboratory Strain 000664) were superovulated and mated at a 1:1 ratio with ~ 15-week-old C57BL/6 J males to generate one-cell zygotes at 0.5 days post-coitum. Fertilised zygotes were harvested, electroporated and transduced essentially as described [28]. In detail, zygotes were washed through 10 droplets of flushing and handling media (FHM) media then added to a mix of equal volumes of Tyrode’s Solution (T1788, Sigma-Aldrich) and FHM for approximately 2–5 s to thin the zona pellucida and transferred immediately to recover in FHM media. The zygotes were then transferred in groups of 20 to 20 μl droplets of KSOM media containing either 1 × 10^9^–1 × 10^11^ rAAV VGC/ml and incubated for 5 h overlayed with sage mineral oil (ART) at 37 °C, 5% CO_2_ and 5% O_2_.

Electroporation was performed using a Nepa 21 (Nepagene) with a 5-mm electroporation chamber (CUY505P5, Nepagene) containing 50 µl Opti-MEM (Thermo Fisher) with 100 ng/µl total sgRNAs and 600 ng/μl Cas9. Four poring pulses were performed at 225 V, with a pulse width of 1 ms and pulse interval of 50 ms at positive polarity, followed by 5 transfer pulses at 20 V, with a pulse length of 50 ms and pulse interval of 50 ms with polarity switching. Pronuclear microinjection of fertilised zygotes was performed with 20 ng/µl Cas9 and 25 ng/µl sgRNA in Microinjection Buffer (10 mM Tris.HCl pH 7.5, 0.1 mM EDTA).

Manipulated zygotes were cultured overnight in rAAV/KSOM to the two-cell stage and surgically reimplanted into recipient pseudopregnant CD1 females, under inhalation anaesthesia (isoflurane) with analgesia provided by pre-operative subcutaneous injection of 0.09 mg/kg buprenorphine (Vetergesic) and 5 mg/kg carprofen (Rimadyl).

### Animal work

All animal studies received ethical approval from the AWERB (Animal Welfare and Ethical Review Body) at the Francis Crick Institute and were performed in accordance with UK Home Office Animals (Scientific Procedures) Act 1986 under project licence PP6551133. Mice were housed in individually ventilated cages and received food and water ad libitum. The health status of the mice was Specific Pathogen Free (FELASA 2004), with the only opportunistic pathogen detected being *Staphylococcus aureus*. For the analysis of gene editing frequencies using live births, no putative founder mice born from the embryo transfers were excluded from the analysis.

### Genotyping founder (F0) mosaic mice

Ear biopsies from founder mice were lysed and initially tested by qPCR using donor integration-specific primer/probe sets (Additional file 1: Table S5). qPCR assays were designed in-house (Integrated DNA Technologies) for all CNV assays and most LOA assays. Two LOA assays (for IRDB and RLEB projects, Additional file 1: Table S5) were designed and conducted by Transnetyx. Either all samples or a subset of integration positive samples were further analysed by qPCR designed against the CRISPR-Cas9 target site, assessing LOA frequency [38] together with a qPCR assay to detect the presence or absence of rAAV vector inverted terminal repeats. Either all samples or a subset of samples were then screened by PCR to assess integration at both the 5′ and 3′ ends of the vector, using GXL polymerase (Takara) with gene-specific primers designed external to regions of homology in combination with primers specific to the targeted allele (Additional file 1: Table S5). PCR amplicons were sequenced by Sanger sequencing (Source Bioscience) or Oxford Nanopore long-read sequencing (Plasmidsaurus) to confirm the integrity of the targeting event.

### Genotyping F1 mice

Selected F0 mice, validated with the above assays were bred to C57BL/6 J mice to achieve germline transmission of the targeted allele in the F1 progeny. F1 mice were then genotyped as described for the F0 founders, but with additional sequencing to ensure 1–1.5 kb on either side of the integration site was captured for a complete analysis of the on-target region via Oxford Nanopore long-read sequencing (Plasmidsaurus).

### Statistical analysis

When comparing proportions between different experimental conditions, e.g. embryo survival, the *z*-test was used to determine significance. When comparing efficiencies on a project (allele basis), a mixed-effect logistic regression model was used. Each result was assigned a set of indicator variables to label which viral titre was used and which gene was targeted. The experimental method was treated as a fixed-effect and the genes as random effects. The model was then fit using the function glmer (family = binomial) from the R package lme4 [39]. The chi-squared test was used to determine the significance of the deviation from the expected Mendelian ratios for the germline transmission data. GraphPad Prism was used for the presentation of data.

### Supplementary Information

**Additional file 1: Table S1 **- Production details for all of the individual embryo manipulation sessions. **Table S2 **- Production details per allele generated, listed by titre of rAAV used. **Table S3 **- Production data for embryo survival and birth rate following delivery by electroporation and pronuclear microinjection, used for the preparation of Fig. 2. **Table S4 **- Animal use dataset used for the presentation of Fig. 6. **Table S5 **- Genotyping primers, qPCR assays, CRISPR sgRNA target sequences and construct details.

## Data Availability

All data generated or analysed during this study are included in this published article and its supplementary information files, with the exception of comparative data from independent studies which was sourced from Table 2 of [25], Table 1 of [30] and Table 1 of [20].

## Abbreviations

DSB: Double-strand break
lssDNA: Long single-stranded DNA
rAAV: Recombinant adeno-associated virus
dsDNA: Double-stranded DNA
ssDNA: Single-stranded DNA
ssODN: Single-stranded oligodeoxynucleotides
LOA: Loss of allele
CNV: Copy number variation
KSOM: Potassium simple optimised media
VGC: Viral genome copy
FHM: Flushing and handling media
RNP: Ribonuclear protein
